# Prevalence and correlates of self-reported and accelerometer-determined sedentary behavior and physical activity of German university students: cross-sectional results of the SmartMoving study

**DOI:** 10.1186/s12889-025-24378-5

**Published:** 2025-09-09

**Authors:** Sascha W. Hoffmann, Jessica Helten, Julika Loss, Claas-Christian Germelmann, Susanne Tittlbach

**Affiliations:** 1 Department of Theory and Practice of Sports and Fields of Physical Activity, Bayreuth Center of Sport Science, University of Bayreuth, Bayreuth, Germany; 2 Department of Social and Health Sciences in Sport, Bayreuth Center of Sport Science, University of Bayreuth, Bayreuth, Germany; 3 Department of Epidemiology and Health Monitoring, Robert Koch Institute, Berlin, Germany; 4 Department of Marketing & Consumer Behavior, University of Bayreuth, Bayreuth, 95440 Germany

**Keywords:** Accelerometry, Physical activity, Sedentary behavior, Active travel, Sleep duration, University students, Health implications

## Abstract

**Background:**

Sedentary behavior (SB) and the absence of physical activity (PA) have become increasingly prevalent in modern societies due to changes in physical and social-environmental conditions, particularly in university students. This cross-sectional study aimed to describe and identify the prevalence and correlates of self-reported and accelerometer-determined SB and PA of German university students.

**Methods:**

A convenience sample of 532 students participated in a questionnaire survey during the lecture period in the summer term 2018. Self-reported total PA, university-, travel-, and leisure-time-related PA were calculated, in addition to self-reported sitting time (ST) within the university. A sub-sample of forty-six participants also received body composition measurements and then wore an ActiGraph wGT3X-BT for seven consecutive days to objectively determine SB, PA, and sleep duration. Differences in descriptive characteristics between samples were analyzed using independent t-test for normally distributed variables or the nonparametric or Mann–Whitney U-Test for not normally distributed variables. Forward stepwise logistic regression analyses were used to analyze correlates associated with self-reported SB and PA in the university context. Stepwise, multiple linear regression analysis was used to determine the associations of anthropometric, sociodemographic, study-related, self-reported SB and PA variables with objectively-determined SB controlling for potential confounders.

**Results:**

Self-reported ST within the university was 2020.5 min/week (95% CI: 1915.8–2125.1), while females reported a 4 h per day higher ST compared with male students (*p* = 0.023). Self-reported PA exclusively at the university was 211.9 min/week (195.3–228.6) with additional time in active travel (AT) of 266.1 min/week (236.8–295.4). Students with higher amounts of sitting (e.g. in the library or in lectures; *p* < 0.001) and students who had lower time in AT (*p* = 0.023) were more likely to be sedentary in the university context. Furthermore, students with a higher monthly net household income (*p* = 0.043), higher values in university-related PA (e.g. walking between lectures or stair climbing; *p* < 0.001) and students who were also engaged in higher times of AT (*p* = 0.004) were more physically active at the university compared with students in the reference group. Correlates associated with accelerometer-determined SB included light-intensity PA (LIPA; *p* < 0.001), moderate-to-vigorous PA (MVPA; *p* < 0.001), sleep duration (*p* < 0.001), monthly net household income (*p* = 0.006) and total cycling time at the university (*p* = 0.032).

**Conclusion:**

Our sample of university students were highly sedentary, but also very active and met current PA recommendations. Daily LIPA, MVPA and also sleep duration were negatively associated with accelerometer-determined SB. Beyond that, daily AT might be a supporting correlate to reduce SB in university students and should be considered as a key variable in future longitudinal interventional studies on activity-friendly and health promoting university environments.

## Background

Sedentary behavior (SB) is defined as any waking behavior characterized by a low energy expenditure ≤ 1.5 metabolic equivalents of task (METs) while in a sitting, lying or reclining position [1]. Recent evidence suggests that uninterrupted, prolonged SB negatively impacts health and leads to an increased risk of all-cause mortality [2–5]. The health risks associated with SB seem to be somewhat independent of engaging at recommended levels of physical activity (PA) [6], but there is evidence from a meta-analysis that ~ 60 min/day of moderate-to-vigorous PA (MVPA) is required to mitigate the increased risk of all-cause mortality associated with high levels of SB. Indeed, a significantly lower risk (*p* < 0.001) of dying during follow-up (Hazard ratio [HR]: 1.04, 95% confidence interval [CI] 0.99–1.10) was associated with those in the most active quartile (> 35.5 MET-h/week) who also reported the most sitting time (> 8 h/day) than those who were most inactive (< 2.5 MET-h/week) and also sat the least (< 4 h/day; HR: 1.27, 95% CI 1.22–1.30) [7]. Moreover, embracing an active lifestyle yields to primary and secondary preventive effects by reducing the risk of non-communicable diseases (NCDs) such as cardiovascular disease (CVD), diabetes mellitus, some cancers and obesity [5, 6, 8]. However, the majority of studies included participants in middle adulthood and mainly reported data from high-income countries [7]. Yet, these findings cannot be generalized across development contexts due to differences in social, behavioral, and environmental determinants of health [9, 10]. Furthermore, self-reported data indicate that lower-income countries receive fewer minor benefits from PA and face more significant harm from SB for mortality and NCD risks [10, 11].

Despite that, a threshold of 6 to 8 h/day of total SB has been identified for an increased risk of all-cause mortality [2, 4, 12]. In this context, population-based studies reported that total SB in adults typically ranges between 5 and 8 h/day and self-reported SB has increased ~ 1 h/day over the past 10 years [2, 6, 13, 14]. In contrast, findings from studies that used device-based measurements showed that total daily SB in adults may be even higher than previously reported and could be in the range of 7.5 to 11.5 h/day [6, 15, 16]. There is substantial variation in SB based on socio-economic factors and occupational status has been identified as a mediating variable [17, 18]. Hence, people working in the white-collar sector, with higher educational levels, and also university students aged 18–24 years were identified as more likely to have long sitting times [17, 19].

Latest estimates of self-reported total SB in German university students were 7 h 25 min, with nearly 50% of the students sitting at least 8 h per weekday [20], whereas a recent meta-analysis reported about accelerometer-determined SB in university students of 9.8 h/day, respectively [21]. This is higher than the global average and even higher compared with levels of SB in white-collar workers, typically involved in long periods of sitting [17, 22]. University students are at high risk of accumulating high levels of SB, due to typical student activities such as attending lectures or seminars as well as studying and learning in the library likely include long periods of uninterrupted, prolonged sitting [20, 21, 23]. High volumes of total sitting time (ST) are omnipresent in the university context and are an integral part of the university culture [20, 21, 24]. Since the transition from adolescence into adulthood is a vulnerable period during which many future life behaviors are established, the university years might also be an important period for the development of future health-related behavior patterns [20, 21, 25]. In this critical period, students are potentially vulnerable to risky health behaviors such as physical inactivity, gaining weight, or to mental health problems and unfavorable sleep habits [26–29]. Therefore, modifiable health-related factors play a crucial role in the context of health promotion of university students and could also be beneficial for the general society since university students are the leaders and decision-makers of tomorrow [20, 24].

A better understanding of the prevalence as well as the correlates of SB and PA among university students could inform the development of future interventions and health policies for this sub-population, and also for the general public [20, 24, 30, 31]. In this context, we recently found out that interrupting prolonged sitting, e.g. with walking of light-intensity, has significant positive effects on certain inflammatory and cardiometabolic risk markers in university students with overweight and obesity [32, 33]. Despite the growing body of research that has analyzed health-related behaviors of university students worldwide over the past years [15, 21, 26, 29, 34–39] and the increasing number of studies examining SB and PA of university students in Germany [20, 24, 25, 40–45], there is still a lack of evidence regarding the prevalence and potential correlates of SB and PA in German university students [20]. To the best of our knowledge, no study has investigated the association of study-related behaviors like sitting in lectures, walking between lectures, and active travel (AT) on SB and PA supported by accelerometer-determined SB and PA in German university students. Therefore, the aim of the present study was to describe and identify the prevalence and potential correlates of self-reported and accelerometer-determined SB and PA within and outside the university setting in a sample of German university students.

## Methods

### Study design

SmartMoving was a two-center cross-sectional study that was undertaken at two German campus universities with total numbers of ~ 13,500 and ~ 21,000 students enrolled assessing (1) SB and PA of German university students, (2) personal barriers and requirements for and against PA, and (3) the efficiency and sustainability of participatory interventions in the university setting. The present study aimed to examine the prevalence and potential correlates of self-reported SB and PA in a convenience sample of German university students. Additionally, we present comprehensive data of accelerometer-determined SB and PA volumes as well as body composition (BC) parameters collected from a sub-sample. A detailed description of the study as well as preliminary results were published elsewhere [40, 41]. The local ethics committee of the University of Bayreuth (Germany) approved the study protocol (O 1305/1-GB) and this trial was planned and carried out in accordance with the principles of Good Clinical Practice and the Declaration of Helsinki [46]. Participation in this study was voluntary, and all participants provided written informed consent before any study related activities were performed.

### Study population and eligibility criteria

**Fig. 1:**
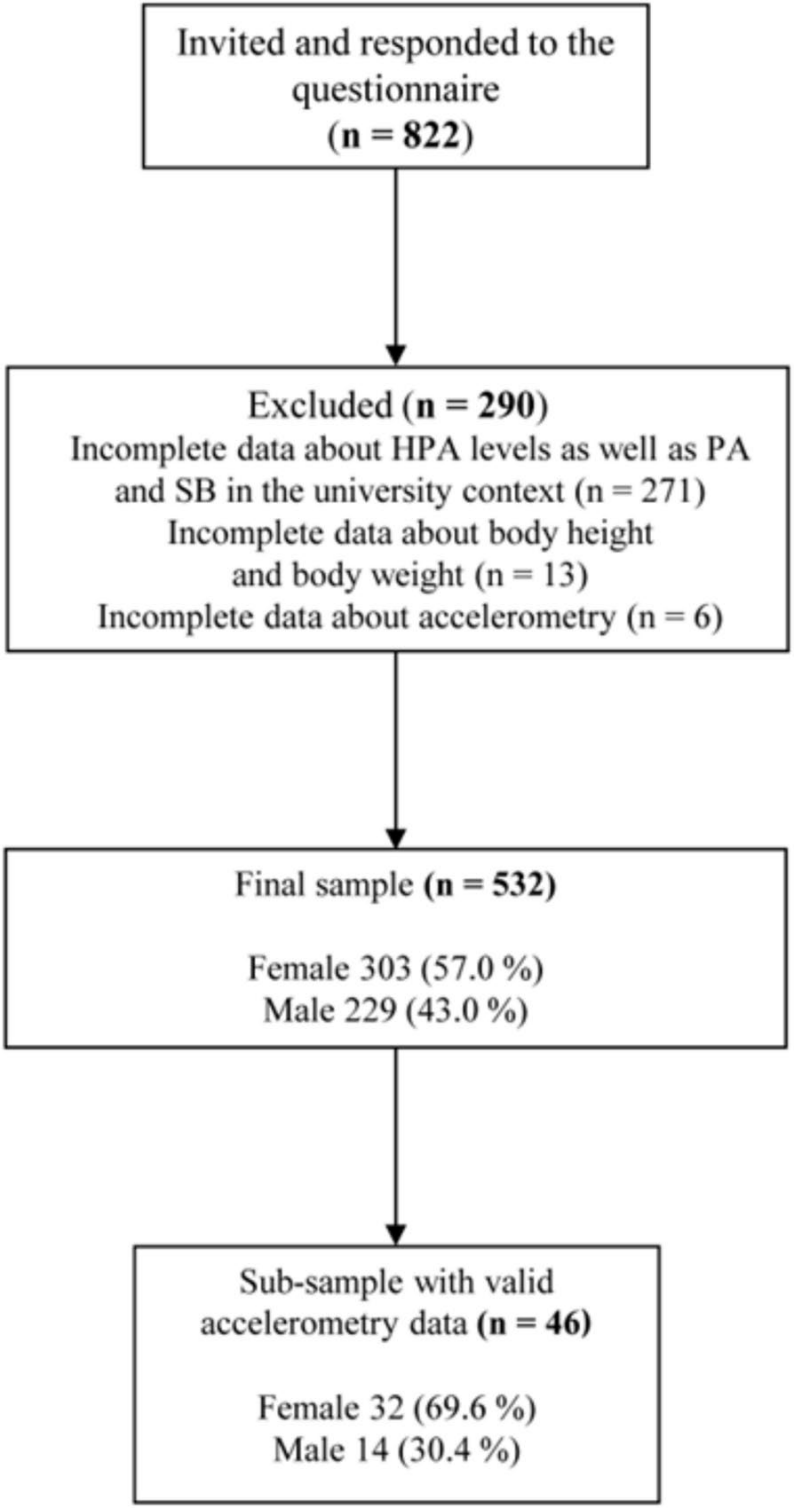
Participant flow chart. HPA, habitual physical activity; PA, physical activity; SB, sedentary behavior

A convenience sample of university students was recruited from two German universities before, during, and after relevant lectures during the regular lecture period of the summer term in 2018. At the beginning of each lecture, the trained staff informed the participants about the aims and the procedures of the study. The participants provided written informed consent and completed a self-administered paper and pencil questionnaire directly in the lecture hall. Respondents placed the completed questionnaire in a box for pickup by project staff at the entrances and exits of the lecture halls. Participants were included in the total study sample if they were ≥ 18 years of age and enrolled in one of the two universities. At the end of the questionnaire, the students were asked whether they would like to participate in a sub-sample to provide accelerometer-determined SB and PA levels, and BC measurements using a bioelectrical impedance analysis (BIA). Therefore, a second written informed consent was required by the participants. Exclusion criteria for the BIA included any implanted device, cardiac arrhythmia, and females who were pregnant or intended to become pregnant. As shown in the study flow chart (Fig. 1), 822 students responded to the questionnaire, 250 participants had incomplete data derived from the PA Questionnaire, while 21 participants did not completely respond to the domain-specific questions of SB and PA within the university setting. A further 13 participants had incomplete information about body height and body weight. Of the 54 participants of the sub-sample, 6 participants did not meet the required wear-time criteria as at least 10 h/day of valid wear time on at least 4 days (including at least one weekend day) and had incomplete accelerometer data as well as missing information about some items from questionnaire. Thus, the final sample consists of 532 participants, 46 of whom belonged to a sub-sample with objectively assessed SB and PA data.

### Self-reported sedentary behavior and physical activity

Questions related to anthropometric and sociodemographic characteristics addressed the student´s age, gender, body height and weight, study semester, field of study, and the net household income available after deduction of rent and health insurance. Self-reported habitual PA (HPA) levels were assessed using a modified Baecke Questionnaire (BAQ) [47, 48]. The validity of the BAQ for the assessment of HPA in different population groups has been reported previously [48, 49]. The questionnaire contains 16 items which can be divided into three meaningful factors. The first factor relates to HPA in the workplace environment, the second factor assessed PA during leisure-time, and the third factor evaluated sport during leisure-time. The participants had to reply on a five-point Likert scale (1 = never, 2 = seldom, 3 = sometimes, 4 = often, 5 = very often or always). Three indices were calculated and incorporated into the analyses: the work index, the leisure-time index, and the sport index. According to the original publication, the test–retest reliability of the work, sport, and leisure-time indices was 0.88, 0.81, and 0.74, respectively [47]. Regarding the work index, there were some small modifications in the formulations of some items to reflect PA levels within the university context [48]. Furthermore, detailed and domain-specific SB and PA (incl. active travel, AT) were assessed using questions from the German PA, Exercise, and Sport Questionnaire (BSA-Questionnaire), and of the ´Sitting Questionnaire´ that measures specific ST in different domains in which the questions were adapted to the university setting. Both questionnaires show significant validity (r = 0.34–0.69) and test–retest reliability (r = 0.78–0.84) [50, 51], but may be less reliable and valid in individuals with lower education levels, the elderly, and in some nationalities [52, 53]. Participants were required to answer the following questions about PA in the university context: “What is the frequency (number of days) and duration (minutes per day) you practice the following PA during your daily life at the university (between or after lectures or while studying etc.)?” The students were allowed to answer regarding the following situations: (1) Walking time to the canteen; (2) going by bike to the canteen; (3) walking time between lectures; (4) going by bike between lectures; (5) going for a walk at the university; and (6) stair climbing at the university. Furthermore, the questionnaire consists of domain-specific questions, assessing the frequency (number of days) and duration (minutes per day) spent on AT (walking, or cycling) in a typical university week during the lecture period. Questions about domain-specific ST were also adapted to the university setting and assessed four different items on weekdays and the weekend during a typical week within the lecture period: The questionnaire prompts participants with the following question: “Please, estimate how long do you normally spend sitting each day in the following situations during a typical week at the university?” Response options were as follows: (1) in the library and learning groups; (2) in lectures; (3) in the canteen; and (4) in breaks (e.g. between lectures). Time spent with PA and with sitting was summed and then multiplied by the number of days the activity was performed (5 for weekdays and 2 for weekend days).

### Objective measurements of sedentary behavior and physical activity

During a separate screening visit an ActiGraph wGT3X-BT (ActiGraph LLC, Pensacola, Florida, USA) accelerometer was handed out to each participant to objectively determine SB and PA during the lecture period. Test–retest reliability of the ActiGraph accelerometer has been previously assessed in adults and results showed intra-class correlation coefficients of 0.70–0.90 [54]. Each participant wore the accelerometer on the hip of their dominant leg with an elastic waistband for seven consecutive days according to the manufacturer´s specifications and above the iliac crest, including during sleep, removing it only for water-based activities (e.g., showering, swimming). Accelerometer records needed to contain at least four valid days (including at least one weekend day), with a valid day defined as containing at least 600 min (10 h/day) of wear time, according to previous studies [35, 54, 55]. Furthermore, participants kept an activity diary considering participants’ answers about their self-reported daily activities to crosscheck the activity data analyzed from the software for wear time and sleep validation. Initializing the accelerometers and analyzing the raw data were achieved using ActiLife software version 6.13.3 (ActiGraph, Pensacola, Florida, USA), and data was collected at 30 Hz. ActiGraph count-based AGD files were accumulated from GT3X files over a 60-s epoch. We defined non-wear time as intervals of 60 min or more of consecutive zeros, with a 2-min allowable interval of counts greater than zero within the upstream/downstream 30-min consecutive zero counts window [55, 56].The outcome variables were the total PA counts per minute (cpm). According to previous studies, SB was defined as < 150 cpm [55, 57], time spent on light-intensity PA (LIPA) (150–1951 cpm) and time spent on MVPA (min/day, ≥ 1952 cpm) [58, 59]. Total PA was then operationalized as the total number of minutes per day spent sedentary, and in light and moderate-to-vigorous PA, based on above-mentioned cpm-thresholds. Total SB and PA (min/day) was then calculated by summing all minutes that met or exceeded the threshold across valid wear days and averaging this value across days for each participant [31, 55, 60]. Sleep–wake patterns were estimated from raw data collected using the Cole–Kripke and Tudor Locke algorithm defining a sleep period of at least 120 min, a bedtime and wake time definition of five minutes and a maximum sleep period of 960 min [61, 62]. From this classification, we calculated total sleep time for each night [63].

### Body composition measurements

Body composition was evaluated using BIA (Nutriguard MS, Data Input GmbH, Pöcking, Germany). Standardized protocol for BIA included the participants had to lie down for 10 min before undergoing a duplicate baseline measurement of BC according to the manufacturer´s instructions. Body height and waist circumference (WC) were measured manually with a calibrated portable stadiometer (Seca 217, Seca, Hamburg, Germany) and using an inelastic tape (Seca 203, Seca, Hamburg, Germany) according to proposed international proceedings. The measurements took place in a separate seminar room at each university. For BIA, the participants were required to fast for at least 6 h and refrain from strenuous PA for at least 12 h before each visit. Alcohol consumption within 24 h of each visit was also prohibited. The following parameters were analyzed during BIA: body weight, fat mass (FM), lean body mass (LBM), total body water (TBW), body cell mass (BCM), extracellular mass (ECM), phase angle, and basal metabolic rate (BMR).

### Data processing and statistical analyses

The normality of the data distribution was assessed using the Shapiro–Wilk test and the visual inspection of the Q-Q plot. A p-value higher than 0.05 indicated that the data were normally distributed and therefore met the assumption of normality, while a p-value less than 0.05 indicated that the data were not normally distributed, and non-parametric tests were used for subsequent analyses. Gender differences between self-reported descriptive characteristics within the total sample and within the sub-sample, and also between the total sample compared with variables in the sub-sample were analyzed with the independent t-test for normally distributed variables or the nonparametric Mann–Whitney U-Test for not normally distributed variables. Furthermore, gender differences between the accelerometer-determined PA and BC variables were also analyzed using the independent t-test for normally distributed variables or the nonparametric Mann–Whitney U-Test for not normally distributed variables. Data are then presented as arithmetic means (95% confidence interval, CI) and relative frequencies. Considering current scientific findings of sitting for more than 8 h/day being associated with significantly increased mortality risk, time spent sitting was dichotomized into sitting < 8 h/day and sitting at least 8h/day [2, 7, 20, 64]. To identify potential cut-off values for an increased risk of having high ST within the university setting, forward stepwise logistic regression analysis was used to calculate odds ratios (ORs) and the corresponding 95% CI assessing the associations between self-reported ST (Dependent variable; dichotomous outcome sitting < 8 h/day vs sitting at least 8 h/day) and the following explanatory variables with the respective scale in brackets: Age (continuous), gender (categorial), study semester (continuous), field of study (categorial), targeted degree (categorial), monthly net household income (categorial), BMI categories (categorial), self-reported HPA levels (continuous) [47], self-reported ST in different university domains (library, lectures, canteen, breaks; categorial) [50], self-reported daily PA behavior and time of AT (categorial) [51]. Dichotomizing was chosen based on the univariate distribution of the exposure variable to support risk stratification, allowing us to more easily identify subgroups for targeted interventions of further studies and to improve the stability of models [65]. Due to the distribution of the data regarding AT and referring to scientific findings of active traveling of about 20 min/day observing significant associations with mortality or cardiovascular risk [66, 67], the variable AT was dichotomized into AT < 20 min/day and AT at least 20 min/day. Regarding LIPA, recent evidence suggests that daily LIPA is associated with a marked reduction in chronic disease and mortality risk in a dose–response manner. In particular, a reduction in cancer mortality risk has been observed for each additional 30 min/day of LIPA that was not different than the risk reduction for 30 min/day of MVPA [68]. Time spent in LIPA in the university setting was therefore dichotomized into LIPA < 180 min/week and LIPA at least 180 min/week according to the six valid days worn by the accelerometer in the sub-sample and based on current findings that even a minimal amount of LIPA per day underscores the importance of routine lifestyle-embedded PA [68]. A second forward stepwise logistic regression analysis was therefore used to assess the associations between self-reported LIPA in the university setting (Dependent variable; dichotomous outcome daily PA at the university < 180 min/week vs daily PA at the university at least 180 min/week) and also the same above-mentioned explanatory variables. Variables were created as binary variables and the interpretation of the coefficients was related to 1 as the reference category compared with the outcome category coded as 2. Overall model fit of these regression models was Nagelkerke’s R^2^ = 0.460 and R^2^ = 0.645, while Akaike Information Criterion (AIC) and Bayesian information criterion (BIC) were between 319.75 to 373.92 for AIC, and between 413.55 to 467.68 for BIC, respectively. The percentages of accuracy in classification were 87.6% and 83.8%. Stepwise, multiple linear regression analysis was used to determine the associations of anthropometric, sociodemographic, study related, self-reported SB and PA factors with objectively-determined SB controlling for accelerometer wear time, age and gender. Regression diagnostics show a coefficient of determination of R^2^ = 0.998 and were performed with appropriate Durban-Watson-Statistics of 2.18 and also indicated there were no problems with multicollinearity among independent variables (VIF between 1.01 and 1.12). Statistical analyses were conducted using GraphPad Prism Version 8.0.2 (GraphPad Software, Inc., San Diego, United States) and IBM SPSS Statistics 28 (IBM, Armonk, United States). The level of significance was set to *p* < 0.05 (two-tailed).

## Results

Descriptive demographic data on gender, age, BMI-category as well as targeted degree of the total sample (n = 532) and the sub-sample (n = 46) are presented in Table 1. About 57.0% of the students were female and mean age was 21.2 years (95% CI: 21.0–21.4). Self-reported mean BMI was 22.2 kg/m^2^ (95% CI: 21.9–22.4), whereas 11.5% were overweight, and 2.1% were obese. The total sample and the sub-sample differed significantly from each other only regarding the sitting behavior within different university domains (e.g. sitting in the library/canteen, *p* < 0.05). Total ST was not significantly different between these two groups.

**Table 1:** Descriptive characteristics of the total sample and the sub-sample

	**Total Sample**				**Sub-Sample**			
**Characteristics**	**Female**	**Male**	*p*-value*	**Total**	**Female**	**Male**	*p*-value*	**Total**
	**Mean (95% CI)**	**Mean (95% CI)**		**Mean (95% CI)**	**Mean (95% CI)**	**Mean (95% CI)**		**Mean (95% CI)**
Demographics								
N, %	303 (57.0)	229 (43.0)	-	532 (100.0)	32 (69.6)	14 (30.4)	-	46 (100.0)
Age, years^1^	21.3 (21.0–21.6)	21.0 (20.7–21.4)	0.150^b^	21.2 (21.0–21.4)	21.5 (20.6–22.5)	22.3 (21.0–23.6)	0.166^b^	21.8 (21.0–22.5)^c^
Study semester^2^	3.6 (3.3–3.9)	3.3 (3.0–3.6)	0.171^b^	3.5 (3.2–3.7)	3.9 (2.7–5.1)	5.1 (3.1–7.0)	0.092^b^	4.2 (3.2–5.2)^d^
First semester students, n (%)	12 (52.2)	11 (47.8)	-	23 (4.4)	1 (100.0)	0 (0.0)	-	1 (2.2)
Field of study, n (%)								
Mathematics, Physics and Computer Science	2 (20.0)	8 (80.0)	-	10 (1.9)	0 (0.0)	0 (0.0)	-	0 (0.0)
Biology, Chemistry, Pharmacy and Earth Sciences	53 (66.3)	27 (33.7)	-	80 (15.0)	7 (77.7)	2 (22.3)	-	9 (19.6)
Law and Economics	76 (48.4)	81 (51.6)	-	157 (29.5)	3 (50.0)	3 (50.0)	-	6 (13.0)
Linguistics and Literary Studies	16 (76.2)	5 (23.8)	-	21 (4.0)	0 (0.0)	0 (0.0)	-	0 (0.0)
Cultural studies (Psychology, Philosophy, Arts, History, Social Sciences, Educational studies, Sport Science and Sport, Business & Law)	88 (53.0)	78 (47.0)	-	166 (31.2)	14 (70.0)	6 (30.0)	-	20 (43.5)
Engineering Sciences	0 (0.0)	2 (100.0)	-	2 (0.4)	0 (0.0)	1 (100.0)	-	1 (2.2)
Medicine	68 (70.8)	28 (29.2)	-	96 (18.0)	8 (80.0)	2 (20.0)	-	10 (21.7)
Targeted degree, n (%)								
Bachelor	272 (56.5)	209 (43.5)	-	481 (90.4)	26 (70.3)	11 (29.7)	-	37 (80.4)
Master	31 (60.8)	20 (39.2)	-	51 (9.6)	6 (60.7)	3 (39.3)	-	9 (19.6)
Net household income^3, 4^, n (%)								
< 500 €	256 (62.0)	157 (38.0)	-	413 (78.5)	29 (64.4)	9 (35.6)	-	38 (84.4)
500–1000 €	41 (40.6)	60 (59.4)	-	101 (19.2)	2 (33.3)	4 (66.7)	-	6 (13.3)
1001–1500 €	0 (0.0)	6 (100.0)	-	6 (1.1)	0 (0.0)	1 (100.0)	-	1 (2.2)
1501–2000 €	2 (50.0)	2 (50.0)	-	4 (0.8)	0 (0.0)	0 (0.0)	-	0 (0.0)
> 2000 €	1 (50.0)	1 (50.0)	-	2 (0.4)	0 (0.0)	0 (0.0)	-	0 (0.0)
Anthropometrics								
Height, cm	168.3 (167.6–169.0)	183.2 (182.3–184.1)	** < 0.001**^**b**^	174.7 (173.8–175.5)	166.8 (164.7–169.0)	186.4 (182.4–190.3)	** < 0.001**^**a**^	172.8 (169.5–176.0)^d^
Weight, kg	61.1 (60.1–62.2)	76.9 (75.5–78.3)	** < 0.001**^**b**^	67.9 (66.8–69.0)	61.1 (58.7–63.6)	80.0 (74.0–86.0)	** < 0.001**^**a**^	66.9 (63.4–70.4)^d^
BMI, kg/m^2^	21.6 (21.2–22.0)	22.9 (22.5–23.3)	** < 0.001**^**b**^	22.2 (21.9–22.4)	22.0 (21.1–22.9)	23.0 (21.8–24.2)	0.211^a^	22.3 (21.6–23.0)^d^
BMI categories^5^, n (%)								
Underweight	88 (53.0)	78 (47.0)	-	37 (7.0)	3 (75.0)	1 (25.0)	-	4 (8.7)
Normal weight	0 (0.0)	2 (100.0)	-	423 (79.5)	26 (70.2)	11 (29.7)	-	37 (80.4)
Overweight	68 (70.8)	28 (29.2)	-	61 (11.5)	3 (60.0)	2 (40.0)	-	5 (10.9)
Obese	68 (70.8)	68 (70.8)	-	11 (2.1)	0 (0.0)	0 (0.0)	-	0 (0.0)
Self-reported ST and PA behavior in the university context^6^								
Work Index	2.6 (2.5–2.6)	2.6 (2.5–2.6)	0.984^b^	2.6 (2.5–2.6)	2.6 (2.5–2.8)	2.6 (2.3–2.8)	0.586^a^	2.6 (2.5–2.7)
Sport Index	3.1 (3.0–3.2)	3.3 (3.2–3.4)	**0.011**^**b**^	3.2 (3.1–3.3)	3.5 (3.3–3.7)	3.5 (3.1–4.0)	0.601^b^	3.5 (3.3–3.7)^c^
Leisure Index	3.1 (3.0–3.2)	2.9 (2.9–3.0)	** < 0.001**^**b**^	3.0 (2.9–3.1)	3.0 (2.9–3.2)	2.9 (2.6–3.1)	0.224^a^	3.0 (2.8–3.1)^d^
Total BAQ-Score	8.8 (8.7–8.9)	8.8 (8.6–8.9)	0.966^a^	8.8 (8.7–8.9)	9.2 (8.9–9.5)	8.9 (8.3–9.6)	0.480^a^	9.1 (8.8–9.4)^d^
ST in the library, min/week	673.8 (587.1–760.6)	474.7 (389.0–560.4)	**0.001**^**b**^	588.1 (526.1–650.2)	930.6 (615.5–1245.7)	700.0 (183.1–1216.9)	0.526	860.4 (600.0–1120.9)^c^
ST in lectures, min/week	954.8 (866.3–1043.3)	939.0 (837.0–1041.0)	0.570^b^	948.0 (881.4–1014.6)	823.6 (611.5–1035.7)	853.6 (405.7–1301.5)	0.782	832.7 (642.0–1023.4)^d^
ST in the canteen, min/week	259.8 (233.9–285.6)	239.3 (206.0–272.5)	**0.028**^**b**^	250.9 (230.5–271.4)	294.2 (219.4–369.1)	335.0 (132.1–538.0)	0.675	306.6 (230.5–382.8)^c^
ST during breaks, min/week	243.1 (214.5–271.8)	220.5 (179.1–262.0)	**0.003**^**b**^	233.4 (209.3–257.5)	234.4 (154.1–314.7)	259.3 (196.9–321.7)	0.298	242.0 (184.3–299.6)^d^
Total ST, min/week	2131.5 (1990.3–2272.7)	1873.5 (1718.8–2028.2)	**0.023**^**b**^	2020.5 (1915.8–2125.1)	2282.8 (1909.9–2655.7)	2147.9 (1350.4–2945.4)	0.715^a^	2241.7 (1904.2–2579.3)^d^
Total walking time, min/week	209.1 (185.6–232.6)	188.1 (164.6–211.7)	0.057^b^	200.1 (183.3–216.9)	168.3 (100.7–236.0)	147.6 (77.7–217.6)	0.990	162.0 (111.8–212.2)^d^
Total time cycling, min/week	11.5 (8.8–14.1)	12.4 (9.5–15.2)	0.264^b^	11.9 (9.9–13.8)	11.2 (3.6–18.9)	7.9 (0.0–18.7)	0.367	10.2 (4.2–16.3)^d^
Total PA, min/week	220.6 (197.2–244.0)	200.5 (177.2–223.8)	0.101^b^	211.9 (195.3–228.6)	179.5 (112.5–246.6)	155.6 (85.8–225.4)	0.747	172.2 (122.4–222.1)^d^
AT, min/week	218.7 (189.8–247.5)	328.7 (273.1–384.3)	0.064^b^	266.1 (236.8–295.4)	181.5 (92.5–270.5)	224.7 (130.1–319.3)	0.181	194.7 (128.2–261.2)^d^
Total PA (incl. AT), min/week	439.3 (399.6–479.0)	529.2 (467.1–591.4)	0.165^b^	478.0 (442.9–513.1)	361.0 (251.6–470.4)	380.3 (272.2–488.4)	0.322	366.9 (286.3–447.5)^d^

Data are presented as means (95% CI), unless otherwise noted

*BMI* body mass index, *BAQ* Baecke Questionnaire, *ST* sitting time, *PA* physical activity, *AT* active travel

*p-values for gender differences were calculated using ^a^independent t-test or ^b^Mann-Whitney U-Test with significant p-values in bold < 0.05. ^1^N = 526 (Total sample); ^2^N = 528 (Total sample); ^3^Net income per month after deducting rent and health insurance; ^4^N = 45 (Sub-sample); ^5^BMI categories according to WHO (2000);^6^self-reported habitual physical activity level derived from the Baecke Questionnaire (Baecke et al. 1982), self-reported sitting behavior in different university situations (library, lectures, cateen, breaks) derived from a modified Sitting Questionnaire (Marshall et al., 2010), self-reported daily physical activity behavior and active travel derived from the BSA-Questionnaire (Fuchs et al., 2015).^c^p < 0.05 for Mann–Whitney U-Test for total values of the entire sample compared to the sub-sample. ^d^p < 0.05 for independent t-test for total values of the entire sample compared to the sub-sample

### Prevalence and correlates of self-reported sitting time and physical activity of the total sample

Self-reported total ST in the university setting was 2020.5 min/week (95% CI: 1915.8–2125.1), while females reported a 4 h/day higher ST compared with male students. This is also true for ST between females and males in the library, in the canteen, and during breaks, respectively. Total PA, e.g. light-intensity walking and cycling, exclusively at the university was 211.9 min/week (95% CI: 195.3–228.6), while additional time in AT was 266.1 min/week (95% CI: 236.8–295.4). Table 2 demonstrates the results of the forward stepwise logistic regression analysis providing correlates associated with self-reported ST of university students in the university setting. Variables were created as binary variables and the interpretation of the coefficients was related to 1 as the reference category. Only significant associations were displayed and the overall model fit was Nagelkerke’s R^2^ = 0.460. Participants who sat for longer periods of time in the library (< 300 min/week = reference; B = 2.36; OR: 10.6; 95% CI: 4.8–23.7, *p* ≤ 0.001), in lectures (< 1000 min/week = reference; B = 2.49; OR: 12.1; 95% CI: 5.6–26.1, *p* ≤ 0.001), in the canteen (< 210 min/week = reference; B = 1.06; OR: 2.9; 95% CI: 1.4–5.8, *p* = 0.003) or during breaks (< 160 min/week = reference; B = 0.89; OR: 2.4; 95% CI: 1.3–4.7, *p* = 0.007) were more likely to be sedentary in the university setting. Compared with students who had lower time in AT (< 20min/day = reference; B = 1) students who engaged in higher amounts of daily AT (B = −0.767; OR 0.5; 95% CI: 0.2–0.9, *p* = 0.032) were less sedentary.

**Table 2:** Correlates associated with self-reported sitting time in the university setting (*n* = 520)

Variables^1^	B^1^	SE^1^	Wald	*p*-value	OR^2^	95% CI
Sitting in the library	2.364	0.409	33.485	< 0.001	10.6	4.8–23.7
(< 300 min/week = reference)					1.00	
Sitting in lectures	2.490	0.394	40.038	< 0.001	12.1	5.6–26.1
(< 1000 min/week = reference)					1.00	
Sitting in the canteen	1.058	0.354	8.943	0.003	2.9	1.4–5.8
(< 210 min/week = reference)					1.00	
Sitting during breaks	0.890	0.331	7.244	0.007	2.4	1.3–4.7
(< 160 min/week = reference)					1.00	
Active travel to the university	−0.767	0.358	4.590	0.032	0.5	0.2–0.9
(< 20 min/day = reference)					1.00	

^1^Only significant associations are displayed. B, slope; SE, unstandardized regression coefficients and standard errors. Variables were created as binary variables and the interpretation of the coefficients was related to 1 as the reference category compared to the outcome category coded as 2. Overall model fit was Nagelkerke’s R2 = 0.460

^2^Odds ratio (OR) and 95% confidence intervals (CI) with p-values (significance at p < 0.05) are from a forward stepwise logistic regression analysis with the following variables included in the regression model: Age, gender, study semester, field of study, targeted degree, monthly net household income, BMI categories, self-reported habitual physical activity level derived from the Baecke Questionnaire (BAQ; Baecke et al. 1982), self-reported sitting behavior in different university situations (library, lectures, canteen, breaks) derived from a modified Sitting Questionnaire (Marshall et al., 2010), self-reported daily physical activity behavior and time of active travel derived from the BSA-Questionnaire (Fuchs et al., 2015)

Table 3 shows correlates associated with self-reported PA in the university setting. Variables were also created as binary variables and the interpretation of the coefficients was related to 1 as the reference category. Only significant associations were displayed, and the overall model fit was Nagelkerke’s R^2^ = 0.645. Students who often walk (< 16 min/week = reference; B = 0.911; OR 2.5; 95% CI: 1.5–4.2, *p* < 0.001) or cycle (< 9 min/week = reference; B = 1.536; OR 4.6; 95% CI: 2.3–9.2, *p* < 0.001) to the canteen, walk between lectures (< 25 min/week = reference; B = 1.658; OR 5.2; 95% CI: 3.0–9.2, *p* < 0.001) or go for a walk (< 60 min/week = reference; B = 3.838; OR 46.4; 95% CI: 24.3–88.8, *p* < 0.001) as well as climbing stairs at the university (< 21 min/week = reference; B = 1.620; OR 5.1; 95% CI: 2.9–8.8, *p* < 0.001) were more likely to have higher PA values within the university setting. Furthermore, students who have higher amounts of daily AT (B = 0.909; OR 2.5; 95% CI: 1.3–4.6, *p* = 0.004) compared with students who had lower time in AT (< 20min/day = reference; B = 1) were more likely to be physically active within the university setting. Finally, students with a higher monthly net household income (< 500 € = reference; B = 0.662; OR 1.9; 95% CI: 1.0–3.7, *p* = 0.043) were also more physically active at the university compared with students in the reference group.

**Table 3:** Correlates associated with self-reported physical activity in the university setting (n = 520)

Variables^1^	B^1^	SE^1^	Wald	*p*-value*	OR^2^	95% CI
Monthly net household income	0.662	0.328	4.076	0.043	1.9	1.0–3.7
(< 500 € = reference)					1.00	
Walking to the canteen	0.911	0.267	11.616	< 0.001	2.5	1.5–4.2
(< 16 min/week = reference)					1.00	
Cycling to the canteen, min	1.536	0.351	19.174	< 0.001	4.6	2.3–9.2
(< 9 min/week = reference)					1.00	
Walking between lectures, min	1.658	0.285	33.944	< 0.001	5.2	3.0–9.2
(< 25 min/week = reference)					1.00	
To go for a walk at the university, min	3.838	0.331	134.813	< 0.001	46.4	24.3–88.8
(< 60 min/week = reference)					1.00	
Stair climbing at the university, min	1.620	0.283	32.723	< 0.001	5.1	2.9–8.8
(< 21 min/week = reference)					1.00	
Active travel to the university, min	0.909	0.314	8.400	0.004	2.5	1.3–4.6
(< 20 min/day = reference)					1.00	

^1^Only significant associations are displayed. B, slope; SE, unstandardized regression coefficients and standard errors. Variables were created as binary variables and the interpretation of the coefficients was related to 1 as the reference category compared with the outcome category coded as 2. Overall model fit was Nagelkerke’s R2 = 0.645

^2^Odds ratio (OR) and 95% confidence intervals (CI) with *p-values (Significance at p < 0.05) are from a forward stepwise logistic regression analysis with the following variables included in the regression model: Age, gender, study semester, field of study, targeted degree, net household income, BMI categories, self-reported habitual physical activity level derived from the Baecke Questionnaire (BAQ; Baecke et al. 1982), self-reported sitting behavior in different university situations (library, lectures, canteen, breaks) derived from a modified Sitting Questionnaire (Marshall et al., 2010), self-reported daily physical activity behavior and time of active travel derived from the BSA-Questionnaire (Fuchs et al., 2015)

### Prevalence and correlates associated with accelerometer-determined sedentary behavior, physical activity, and body composition parameters of the sub-sample

Table 4 presents accelerometer-determined PA and BC parameters of the sub-sample separated by gender. The average value of objectively determined SB was 10.4 h/day (95% CI: 9.9–10.8), and the average values of LIPA and MVPA were 4.3 h/day (95% CI: 3.9–4.6), and 1.1 h/day (95% CI: 0.9–1.1), respectively. Daily sleep duration was about 8.2 h/day, while daily wear time was 20.0 h/day. Overall, we observed no significant gender differences regarding these PA values and basal anthropometry showed no abnormal values.

**Table 4:** Accelerometer-determined physical activity and body composition parameters of the sub-sample (n = 46)

**Characteristics**	**Total**	**Female**	**Male**	*p-*value*
	Mean (95% CI)	Mean (95% CI)	Mean (95% CI)	
N	46 (100.0)	32 (69.6)	14 (30.4)	-
Physical Activity^1^				
SB, h/day	10.4 (9.9–10.8)	10.5 (10.1–11.0)	9.9 (8.8–11.1)	0.265^a^
LIPA, h/day	4.3 (3.9–4.6)	4.1 (3.8–4.5)	4.7 (4.0–5.4)	0.073^a^
MVPA, h/day	1.1 (1.0–1.3)	1.1 (1.0–1.2)	1.3 (1.0–1.5)	0.187^a^
MPA, h/day	1.0 (0.9–1.1)	1.0 (0.9–1.1)	1.1 (0.9–1.3)	0.305^a^
VPA, h/day	0.1 (0.0–0.1)	0.1 (0.1–0.1)	0.1 (0.0–0.2)	0.990^b^
Sleep, h/day	8.2 (7.9–8.5)	8.3 (7.9–8.6)	8.0 (7.4–8.6)	0.415^a^
Step counts, no/day	9936.9 (9130.4–10,743.4)	9653.3 (8726.3–10,580.3)	10,585.1 (8841.4–12,328.7)	0.289^a^
Wear time, h/day	20.0 (19.7–20.3)	20.1 (19.8–20.5)	19.8 (19.0–20.5)	0.228^b^
Valid days worn, days	6.0 (5.8–6.2)	6.1 (5.9–6.2)	5.9 (5.6–6.2)	0.150^b^
Body Composition				
BMI, kg/m^2^	22.3 (21.6–23.0)	22.0 (21.1–22.9)	23.0 (21.8–24.2)	0.211^a^
FM, kg	11.4 (10.1–12.7)	12.5 (11.0–14.0)	8.9 (7.0–10.8)	**0.006**^**a**^
FM, %	17.1 (15.2–19.0)	19.8 (18.0–21.7)	10.9 (8.8–13.0)	-
TBW, L	41.1 (38.5–43.7)	36.2 (34.8–37.5)	52.2 (48.5–55.9)	** < 0.001**^**b**^
LBM, kg	56.1 (52.6–59.6)	49.5 (47.7–51.2)	71.3 (66.2–76.4)	** < 0.001**^**b**^
BCM, kg	32.2 (29.8–34.6)	27.5 (26.4–28.7)	42.8 (39.7–45.9)	** < 0.001**^**b**^
ECM, kg	23.9 (22.7–25.2)	21.9 (21.1–22.7)	28.5 (26.1–30.9)	** < 0.001**^**b**^
BCM in LBM, %	57.0 (56.1–57.9)	55.7 (54.8–56.5)	60.1 (58.6–61.5)	-
Phase angle, °	7.2 (7.0–7.2)	6.8 (6.6–7.0)	8.0 (7.6–8.4)	** < 0.001**^**a**^
WC, cm	75.9 (73.5–78.4)	72.4 (70.1–74.7)	84.1 (80.5–87.6)	** < 0.001**^**a**^
BMR, kcal	1633.3 (1557.2–1709.3)	1486.9 (1449.7–1524.0)	1967.9 (1870.3–2065.5)	** < 0.001**^**a**^

Data are presented as means (95% CI), unless otherwise noted

*SB* sedentary behavior, *LIPA* light-intensity physical activity, *MPA* moderate physical activity, *MVPA* moderate-to-vigorous physical activity, *VPA* vigorous physical activity, *BMI* body mass index, *FM* fat mass, *TBW* total body water, *LBM* lean body mass, *BCM* body cell mass, *ECM* extracelluar mass, *WC* waist circumference, *BMR* basal metabolic rate

*p-values were calculated using ^a^independent t-test or ^b^Mann-Whitney U-Test with significant p-values in bold <0.05. ^1^Physical activity was measured with the ActiGraph wGT3X-BT accelerometer. Data are presented as means (95% CI), unless otherwise noted

Table 5 shows significant correlates associated with accelerometer-determined SB derived from a stepwise multiple linear regression analysis and revealed that students with higher daily LIPA (β = −0.684; *p* < 0.001) and MVPA (β = −0.250; *p* < 0.001) as well as students who slept longer per night (β = −0.658; *p* < 0.001) were less likely to be sedentary. This is also true for students with a higher monthly net household income (< 500 € = reference; β = −0.016; *p* = 0.006) and for students with higher total cycling time at the university (β = −0.012; *p* = 0.034) who were less sedentary.

**Table 5:** Correlates associated with accelerometer-determined sedentary behavior (*n* = 46)

Variables^1^	β	B (95% CI)	*p*-value*
LIPA, h/day	−0.684	−0,980 (−0.996, −0.963)	< 0.001
MVPA, h/day	−0.250	−1,012 (−1.015, −0.965)	< 0.001
Sleep, h/day	−0.658	−0,998 (−1.015, −0.982)	< 0.001
Monthly net household income (< 500 € = reference)	−0.016	−0,066 (−0.111, −0.020)	0.006
Total cycling time at the university, min/week	−0.012	−0,001 (0.000, −0.002)	0.034

^1^Only significant associations are displayed. The variable"Monthly net household income"was created as a binary variable and the interpretation of the coefficients was related to 1 as the reference category compared with the outcome category coded as 2. Overall model fit was R^2^ = 0.998. Variables derived from a stepwise multiple linear regression analysis: Age, gender, study semester, field of study, targeted degree, net household income, BMI categories, self-reported habitual physical activity level derived from the Baecke Questionnaire (BAQ; Baecke et al. 1982), self-reported sitting behavior in different university situations (library, lectures, cateen, breaks) derived from a modified Sitting Questionnaire (Marshall et al., 2010), self-reported daily physical activity behavior and active transport time derived from the BSA-Questionnaire (Fuchs et al., 2015)

**Fig. 2:**
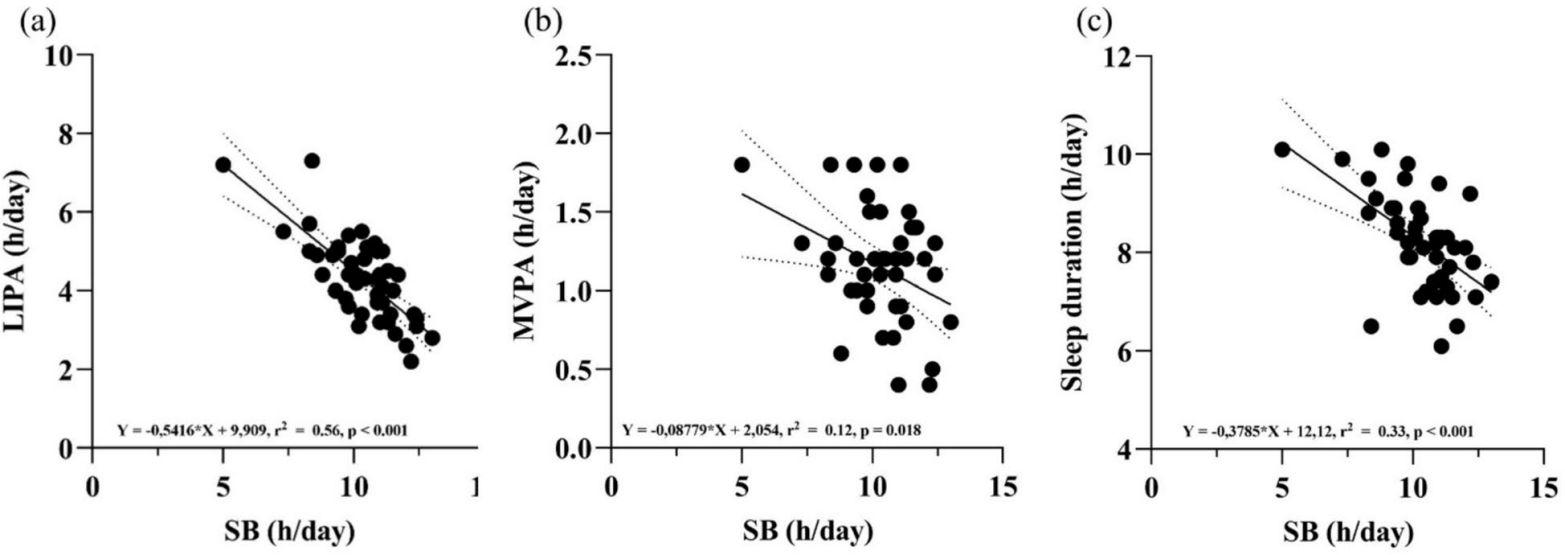
Simple linear regression analyses with the respective regression lines (bold lines) and the 95% CIs (dotted lines) between accelerometer-determined sedentary behavior (SB) and PA variables, and sleep duration. **a** Light-intensity physical activity (LIPA) and SB; (**b**) Moderate-to-vigorous physical activity (MVPA) and SB; (**c**) Daily sleep duration and SB

Figure 2 displays simple linear regression analyses between accelerometer-determined PA values and SB. Regarding the association between sleep duration and SB (h/day), the respective slope of the regression line indicates that a 100 min/day higher SB level was associated with a lower sleep duration of −37 min/day, for example. Furthermore, an increase in daily SB of 100 min/day was associated with a decrease in daily LIPA of 54 min/day, respectively.

## Discussion

University students are at high risk of having high amounts of SB and further unhealthy lifestyle behaviors, like being overweight or having poor mental health [20, 26, 35, 69]. To the best of our knowledge, this is the first study that (1) examined the prevalence and identified potential correlates of self-reported and also accelerometer-determined SB and PA in German university students; and (2) provided comprehensive information about detailed sitting times of university students in different domains within the university setting.

Our study extends previous research by quantifying SB and PA in university students using self-report measurement tools [20, 21, 26, 70] and tri-axial accelerometry [15, 34–36, 39, 60] to obtain comprehensive information about the prevalence and correlates of SB and PA in this specific sub-group. To date, only one study of our research group recently objectively determined SB and PA volumes as an ancillary measure in a small sample of German university students with overweight and obesity and revealed that the participants spent about two third (~ 57 h/week) of their time during the week sedentary, almost on third (~ 27 h/week) in LIPA, and 6.5% (~ 5 h/week) performing MVPA, respectively [71].

### Sedentary behavior

Accompanied by the data from the accelerometry, total SB in university students in the present study was 602 min/day (10 h 2 min). This is 12 min higher (9 h 50 min) than the total amounts of objectively-measured SB, and 2 h 43 min (7 h 29 min) higher compared to self-reports obtained from a recently published meta-analysis, with almost one third of the reviewed studies carried out at European universities [21]. Similar to research findings in the field, objectively assessed data on SB have shown to be even higher than self-reported information [72, 73]. However, our results are in line with results from a study from the United States, presenting similar average times per day spent in SB (10.0 ± 1.2 h) in a sample of university students 18–20 years of age [34]. Our participants accumulated slightly higher accelerometer-determined SB than the observed young adults aged 22 ± 0.6 years obtained from the Raine study cohort in Australia (9.2 ± 1.6 h/day, 61.4%) [15]. This is also true for the comparison with data from Spain, where the accelerometer-determined mean time students spent in SB was ~ 10 h/day [39]. Even higher SB (> 12 h/day, resulting in > 80% waking hours) were recorded in a sample of university students from the United Arab Emirates [36].

Regarding the detailed sitting times in different domains within the university setting, the overall self-reported ST exclusively at the university was 4 h 49 min per day and almost similar compared with data from the accelerometry. In addition, students spent 2 h 18 min and 1 h 26 min sedentary in lectures and in the library, respectively. The average adult (> 18 years) in Germany usually sits for 5 h per day whereas the age peers in Germany (18–29 years old) were sitting on average 6 h 9 min day [17, 74]. The present data demonstrate that our sample of university students probably sit longer compared with their age peers in Germany and also have comparable sitting times of desk workers sitting on average about 7 h per day [17, 20, 43, 74].

A statistically significantly higher risk or all-cause mortality was observed from 6 to 8 h of self-reported ST per day, or 9.5 or more hours auf accelerometer-assessed ST per day [2, 16]. Our results pointed out that 14.7% of our students reported sitting times at least 8 h per day exclusively at the university and are possibly exposed to an increased risk of premature mortality. In contrast to prior research, female students in our sample had significantly longer ST in almost all domains in the university setting, with except sitting in lectures [20]. These findings are consistent with others in which young women spent about 30 to 60 min more per day in sedentary time than young men [15, 75].

Furthermore, our results provided correlates associated with student`s self-reported high ST within the university setting. The risk of having high ST within the university already increases at low daily thresholds of sitting in lectures or in the library, which in turn are typical activities associated with studying [21, 76, 77]. Interestingly, daily AT of equal or less than 20 min/day significantly interact with student`s ST during their stay at the university. Although PA as an intrapersonal variable has been identified as a potential correlate of SB in university students, the now mentioned AT as an influencing factor in student`s SB is new and may provide future research approaches on modifiable correlates covering further socio-ecologic levels [69]. Results of a recently published review revealed that there is a growing body of research indicating inverse associations between AT and diverse health parameters, such as cardiovascular diseases, cancer, mental or self-rated health as well as physical fitness [78]. Therefore, focusing on increased AT in university students may have the chance to (1) counteract high ST in the university setting, (2) contribute to improve student`s health and (3) be also beneficial for the health of society from a public health and sustainability perspective. Based on these findings, intervention models promoting AT need to be established within a university and student health management and possibly become a basic task of universities from a public health perspective in the near future.

### Physical activity

Overall, the PA analysis revealed that our sample of university students spent most of their activity counts in LIPA, resulting in 4 h 14 min per day. Total self-reported LIPA exclusively within the university setting (e.g. walking or cycling) was 30 min per day with additional time of 38 min per day of AT, while time in MVPA was about 60 min per day. Thus, our study participants were highly sedentary but also very active and met current PA recommendations of 150 min per week of MVPA [8, 79]. The average time spent in MVPA by our study participants was similar to findings by Peterson et al. [34], and Vella et al. [31], who also examined young university students, whereas time in LIPA was about 1 h 34 min lower in our sample compared to the above-mentioned studies. This is also true for the comparison of BC parameters showing similar but slightly lower values for FM and WC, particularly in women. In contrast, daily mean MVPA of our sample was almost double as high compared with age peers from Australia (33 min/day) [15], and Canada (29 min/day) [80]. Recent research from Germany, in turn, indicated that 22.4% of the sample of university students were assigned as insufficiently active [20]. Further research on 18 to 29 years old persons in Germany found a proportion of 43% not meeting national recommendations on PA [79, 81]. University students therefore seem to be more active than their age peers in the German population. Due to their time in MVPA of approx. 1 h/day, university students may further have the chance to counteract or eliminate the negative effects of uninterrupted, prolonged sitting and possibly reduce their individual mortality risk associated with high SB > 8 h/day [6, 7]. In this context and as we recently pointed out, integrating light-intensity PA snacks (LIPAS) seem to positively influence markers associated with inflammation and cardiometabolic importance in this young adult population [32, 33, 71]. Moreover, higher levels of LIPA are also associated with a substantially reduced risk for premature mortality with maximal risk reductions at about 375 min/day [16]. Our analysis revealed furthermore that students with higher amounts of daily SB had significantly shorter sleep duration per night than their counterparts. Recent evidence suggests that short sleep duration in general has been identified as a risk factor for cardiometabolic disorders [82], and, furthermore, was associated with unfavorable meal consumption habits in university students throughout the day [83]. Thus, the interaction and combination of high SB and short sleep duration in university students and their possible contribution to the public health burden requires further attention in future studies.

We found significant differences between female and male students in self-reported sport and leisure-time indices from the BAQ indicate that female students had lower values in the sport domain but were more physically active during their leisure-time compared with male students in the total sample. Previous research on university students also found that females were less physically active than their male counterparts [20, 84], which aligns also with previous studies of the European and German adult population [81, 85, 86]. There is, however, a greater likelihood among men to overreport PA compared to women [72]. In contrast to previous results from university students [20, 84, 87], our accelerometer-derived PA data revealed no significant gender differences. Regarding possible correlates of self-reported PA in the university setting, our analysis revealed that students with a higher monthly net household income, higher values in university-related PA (e.g. walking between lectures or stair climbing) and students who were also engaged in higher times of AT were more physically active at the university compared with students in the reference group. The literature shows no comparable data from German university students, otherwise a positive association between household income and daily PA in medical students has been observed [88]. Furthermore, another study pointed out that there is a positive influence of parental exercise habits on college students´ PA [89], and that high levels of parental PA are systematically associated with increased levels of PA in their children and until the children reach tea age of 14 [90]. This might also influence our results, since the mean age of our study sample was 21 years. Equivalent to the association between AT and self-reported ST in the university, engaging in higher times of AT lead to more physically active students within the university setting. One study suggested that active travelling decreased the risk of obesity, hypertension and diabetes [67], while a further study pointed out that travelling actively offers an effective way of integrating PA into daily life [66], and an increased PA is identified as the most important determinant of health benefits of AT in adults [91]. There is still not enough research to distinguish what mode of AT is best for health, but cycling appears to be beneficial considering potential health benefits [78]. In addition to these facts, our findings also revealed that total time cycling at the university is negatively associated with objectively-assessed SB. This is in line with recently published research that active travel behaviors in university students are linked to lower objectively measured sedentary time during different commuting segments, and this may reinforce the health value of walking and cycling beyond their contribution to PA and SB metrics [92].

### Strengths and limitations

The strength of this study is that it provides novel insights regarding the reporting of SB and PA of German university students which can be addressed exclusively to different domains within the university setting. Additionally, for the first time, this study provides data of accelerometer-determined SB and PA with accompanying BC measurements in a sub-sample of healthy German university students. The participants were very compliant with the accelerometer protocol showing high wear time per day and providing quality accelerometer data with at least four valid days (including at least one weekend day). However, several limitations should be considered when interpreting our findings. Since our sample was a convenience sample of volunteers, sampling bias may have been introduced in the sense that more active volunteers may have been more likely to participate in a study that monitored physical activity. Females were slightly overrepresented in the present study. The findings of this study are subject to the limitations of respondent self-reporting and its cross-sectional design, which limits our ability to draw conclusions about the associations between exposures and outcomes. Causal relationships are not clear at present. The sample size of our sub-sample was relatively small, and the obtained data and the conclusions of this study should be interpreted carefully for hypothesis generation. Larger studies, particularly with a longitudinal observational design, are needed to draw causal relationships and quantify our findings. Wearing the accelerometers may cause some reactivity by the participants (Hawthorne effect) [39], and it is unknown whether the students conducting the measurements are reliable. Our results might have been different if we had used other algorithms conducting wear time validation and bout counts. Finally, PA levels follow seasonality variations finding higher PA levels on summer-spring months compared with winter. This may have led to an overestimation of average PA levels in our sample and may limit the generalizability of our findings to other seasons.

### Practical implications

The results from the present study support the hypothesis that university students may be at greater risk of high SB levels [17]. In turn, our data revealed that our participants from two German universities were also active and met the current PA recommendations [8]. Based on recommendations from previous research in this population group, for the first time, we evaluated a combination of self-reported and objective accelerometer-determined information to identify potential correlates of SB and PA of university students and within the university setting [20]. Our findings are important as they help to identify previously unnoticed behavior patterns and consequences of inadequate PA and SB levels as well as possible structural barriers to enhance PA and possible reasons for high amounts of SB in German university students.

Given the high levels of SB observed in this study – particularly in university-specific domains such as lectures and study environments – multilevel strategies are warranted to reduce SB and promote PA among students. In addition to the students'own responsibility and their daily challenges by choosing between all kinds of activities, academic institutions should integrate structured physical activity breaks into lectures and provide opportunities for standing or movement during learning sessions, including in libraries and other sedentary-prone settings. The promotion of active travel—especially walking and cycling over distances exceeding 20 min per day—should be prioritized, supported by the expansion of safe infrastructure and the implementation of incentive-based programs (e.g., bike-sharing services or mobility campaigns). To address observed socio-economic disparities, movement-promoting initiatives should be accessible regardless of financial background, for example through subsidized sports programs or equipment rental schemes. Gender-sensitive approaches are recommended, given the higher domain-specific sitting times reported by female students. Moreover, student health promotion efforts should consider the inverse association between SB and sleep duration by integrating messages on sleep hygiene and PA behavior. Overall, the findings support the development of institutional guidelines that embed SB reduction and PA promotion into university policy, curricula, and campus infrastructure. Future intervention studies should evaluate the effectiveness and feasibility of such measures within the higher education context and should examine activity methods in university teaching and possible improvements to the university's infrastructure to encourage PA between inevitable sitting periods.

## Conclusion

In summary, our study showed high levels of PA combined with high amounts of SB in German university students. Regarding SB at the university, the majority of typical student activities, like attending lectures and learning in the library, should be interrupted as often as possible with light-intensity walking, cycling or stair climbing, whereas daily AT might be a supporting correlate in promoting PA and reducing SB in everyday students´ lifecycle. We therefore recommend integrating LIPAS (e.g., walking or cycling) and AT as often as possible into a student’s daily schedule and beyond their studies. Future longitudinal research is needed determining the mechanisms linking LIPAS, AT and sleep to SB and PA of university students in more detail to develop tailored prevention and intervention strategies enhancing an activity-friendly university environment.

## Data Availability

Data will be available upon reasonable request from the corresponding author.

## Abbreviations

AE: Activity expenditure
AIC: Akaike Information Criterion
AT: Active travel
BAQ: Baecke Questionnaire
BC: Body composition
BCM: Body cell mass
BIA: Bioelectrical impedance analysis
BIC: Bayesian information criterion
BMI: Body mass index
BMR: Basal metabolic rate
BSA-Questionnaire: German physical activity, exercise, and sport questionnaire
CI: Confidence interval
Cpm: Counts per minute
CVD: Cardiovascular disease
ECM: Extracellular mass
FM: Fat mass
HPA: Habitual physical activity
LBM: Lean body mass
LIPA: Light-intensity physical activity
LIPAS: Light-intensity physical activity snacks
METs: Metabolic equivalents of tasks
MVPA: Moderate-to-vigorous physical activity
NCDs: Non-communicable diseases
ORs: Odds ratios
PA: Physical activity
Q-Q-plot: Quantile-quantile-plot
R^2^: R-squared/coefficient of determination
SB: Sedentary behavior
ST: Sitting time
TBW: Total body water
VIF: Variance inflation factor
WC: Waist circumference
